# Enhanced AKT Phosphorylation of Circulating B Cells in Patients With Activated PI3Kδ Syndrome

**DOI:** 10.3389/fimmu.2018.00568

**Published:** 2018-04-05

**Authors:** Takaki Asano, Satoshi Okada, Miyuki Tsumura, Tzu-Wen Yeh, Kanako Mitsui-Sekinaka, Yuki Tsujita, Youjiro Ichinose, Akira Shimada, Kunio Hashimoto, Taizo Wada, Kohsuke Imai, Osamu Ohara, Tomohiro Morio, Shigeaki Nonoyama, Masao Kobayashi

**Affiliations:** ^1^Department of Pediatrics, Hiroshima University Graduate School of Biomedical & Health Sciences, Hiroshima, Japan; ^2^Department of Pediatrics and Developmental Biology, Tokyo Medical and Dental University (TMDU), Tokyo, Japan; ^3^Department of Pediatrics, National Defense Medical College, Tokorozawa, Japan; ^4^Department of Pediatrics, Ako Central Hospital, Ako, Japan; ^5^Department of Pediatrics, Okayama University Graduate School of Medicine, Okayama, Japan; ^6^Department of Pediatrics, Nagasaki University Graduate School of Biomedical Sciences, Nagasaki, Japan; ^7^Department of Pediatrics, School of Medicine, Institute of Medical, Pharmaceutical, and Health Sciences, Kanazawa University, Kanazawa, Japan; ^8^Department of Community Pediatrics, Perinatal and Maternal Medicine, Tokyo Medical and Dental University (TMDU), Tokyo, Japan; ^9^Department of Technology Development, Kazusa DNA Research Institute, Kisarazu, Japan

**Keywords:** activated PI3 kinase delta syndrome, AKT phosphorylation, catalytic subunit p110δ of phosphatidylinositol 3-kinase, flow cytometry, immunodeficiency, regulatory subunit p85α of phosphatidylinositol 3-kinase

## Abstract

Activated PI3Kδ syndrome (APDS) is a primary immunodeficiency characterized by recurrent respiratory tract infections, lymphoproliferation, and defective IgG production. Heterozygous mutations in *PIK3CD, PIK3R1*, or *PTEN*, which are related to the hyperactive phosphoinositide 3-kinase (PI3K) signaling, were recently presented to cause APDS1 or APDS2 (APDSs), or APDS-like (APDS-L) disorder. In this study, we examined the AKT phosphorylation of peripheral blood lymphocytes and monocytes in patients with APDSs and APDS-L by using flow cytometry. CD19^+^ B cells of peripheral blood in APDS2 patients showed the enhanced phosphorylation of AKT at Ser473 (pAKT) without any specific stimulation. The enhanced pAKT in CD19^+^ B cells was normalized by the addition of a p110δ inhibitor. In contrast, CD3^+^ T cells and CD14^+^ monocytes did not show the enhanced pAKT in the absence of stimulation. These findings were similarly observed in patients with APDS1 and APDS-L. Among CD19^+^ B cells, enhanced pAKT was prominently detected in CD10^+^ immature B cells compared with CD10^−^ mature B cells. Enhanced pAKT was not observed in B cells of healthy controls, patients with common variable immunodeficiency, and hyper IgM syndrome due to CD40L deficiency. These results suggest that the enhanced pAKT in circulating B cells may be useful for the discrimination of APDS1, APDS2, and APDS-L from other antibody deficiencies.

## Introduction

Activated PI3Kδ syndrome (APDS) is a primary immunodeficiency (PID) characterized by recurrent respiratory tract infections, chronic Epstein Barr virus and cytomegalovirus infections, lymphoproliferation, increased lymphoma susceptibility, and poor antibody production (1–4). Heterozygous gain-of-function mutations in *PIK3CD*, which encodes the catalytic subunit p110δ of phosphoinositide 3-kinase (PI3K), have been identified in patients with APDS1 (1, 3). Subsequent studies have demonstrated that a heterozygous mutation in *PIK3R1* encoding p85α, a regulatory subunit of PI3K, is responsible for APDS2: a PID with similar clinical manifestations to APDS1 (2, 4). Moreover, a patient with a heterozygous loss-of-function mutation in *PTEN*, which encodes phosphatase and tensin homolog and is associated with PTEN hamartoma tumor syndrome (PHTS), was recently reported to develop APDS-like immunodeficiency (APDS-L) with incomplete penetrance (5, 6).

Phosphoinositide 3-kinases convert phosphatidylinositol 3,4-triphosphate (PIP2) to phosphatidylinositol 3,4,5-triphosphate (PIP3) and are involved in cellular functions including proliferation, differentiation, survival, and trafficking (7, 8). Both p110δ and p85α belong to class IA PI3Ks and have an essential role in the differentiation, development, and functions of several distinct stages of B- and T-lymphocytes (7, 8). They also have an important role in the antibody maturation process by regulating immunoglobulin class-switch recombination and plasma cell differentiation (9). When stimulated, PIP3 recruits AKT to the plasma membrane where AKT is activated *via* phosphorylation by PDK1 and mTORC2 (7, 8). In contrast, PTEN antagonizes PI3Ks by catalytic dephosphorylation of PIP3 to PIP2 (10). The recent identification of APDS1 and APDS2 (APDSs) and APDS-L revealed that the hyperactive PI3K/AKT signaling affects the immune system in humans, leading to the development of PID.

Here, we investigated four unrelated Japanese patients with APDS2 caused by a heterozygous mutation in *PIK3R1*. Activated T cells from the patients showed enhanced phosphorylation of Ser473 of AKT (pAKT) consistent with findings in previous reports (2, 4). We next investigated the status of pAKT using peripheral blood mononuclear cells (PBMCs) isolated from patients and analyzed by flow cytometry. We observed that circulating CD19^+^ B cells from APDS2 patients, but not other cell populations, showed enhanced pAKT. This finding was similarly detected in circulating CD19^+^ B cells from patients with APDSs or APDS-L, but not from healthy controls, common variable immunodeficiency (CVID) patients, or hyper IgM syndrome (HIGM) patients due to CD40L deficiency. Therefore, enhanced pAKT signaling in circulating CD19^+^ B cells was considered a specific finding in patients with APDSs or APDS-L. Furthermore, by focusing on CD10^+^CD19^+^ immature B cells, this method allowed us to distinguish APDSs and APDS-L patients from healthy controls and patients with CVID or HIGM (CVID/HIGM). This flow-cytometry-based assay of PI3K activity enabled the discrimination analysis of identified mutations in *PIK3CD, PIK3R1*, or *PTEN*. It may also serve as a rapid diagnostic method to discriminate APDSs and APDS-L patients from other PID.

## Materials and Methods

### Cases

We investigated four unrelated Japanese patients with APDS2 who were involved in a previous international survey (P13, P14, P19, and P26 in the previous report) (11). The detailed clinical manifestations of those patients are available in Materials and Methods in Supplementary Material. All of the patients carried heterozygous germline mutations in *PIK3R1* (Figure S1 in Supplementary Material). The identification of *PIK3R1* mutation was performed by a candidate gene approach in P4. For the other three patients, the mutations were identified by whole exome sequencing and were confirmed by Sanger sequencing. The identified mutations were 1425 + 2 T > A (P1), 1300−1 G > C (P2), 1425 + 1 G > C (P3), and 1425 + 1 G > T (P4). The *PIK3R1* mutations identified in P3 and P4 were previously shown to be pathogenic mutations (2, 4).

We included four CVID patients, aged 33 (P5), 36 (P6), 17 (P7), and 30 (P8) years, whose genetic causes have not been identified (detailed in Materials and Methods in Supplementary Material). The absence of pathogenic mutations in *PIK3CD, PIK3R1*, and *PTEN* was confirmed in those patients. We also included one HIGM patient due to CD40L deficiency (P9) who was 41 years old (detailed in Materials and Methods in Supplementary Material).

### Immunoblot Analysis

CD3^+^ T cells and CD19^+^ B cells were separated from PBMCs using the IMag™ Cell Separation System (BD Biosciences, San Jose, CA, USA). The separated cells were then subjected to immunoblot analysis using the following antibodies: anti-AKT antibody (Cell Signaling Technology, Danvers, MA, USA), anti-phospho-AKT (Ser473) antibody (Cell Signaling Technology), and ß-actin (SIGMA-ALDRICH, Saint Louis, MO, USA).

### Preparation of Activated T Cells

Activated T cells were derived from PBMCs according to a previous report (2). Briefly, PBMCs were cultured with 1 × 10^6^ cells per mL in RPMI 1640 GlutaMax supplemented with 10% human AB serum, penicillin and streptomycin, PMA (1 µmol/L), and ionomycin (20 ng/mL) for 2 days. The cells were then separated by Lymphoprep density-gradient centrifugation and washed twice with RPMI 1640 GlutaMax. Then, they were cultured in RPMI 1640 GlutaMax supplemented with 10% human AB serum and IL-2 (100 IU/mL) for 16–24 h.

### B-Cell Stimulation

For B-cell stimulation, PBMCs were purified by Lymphoprep density-gradient centrifugation and incubated at 1 × 10^6^ cells per mL in RPMI 1640 GlutaMax supplemented with 10% human AB serum, penicillin, and streptomycin. The cells were stimulated with CD40L (1 µg/mL) and IL-4 (20 ng/mL) for 30 min. They were then harvested and subjected to flow-cytometry analysis of AKT phosphorylation.

### Flow-Cytometry Analysis of AKT Phosphorylation

Peripheral blood mononuclear cells from APDS1 (four patients), APDS2 (four patients), APDS-L (four patients), CVID (four patients), HIGM (one patient), and 24 adult healthy controls were subjected to flow-cytometry analysis. We assessed pAKT at Ser473 by flow cytometry as follows. PBMCs were suspended at a density of 1 × 10^6^ cells/μL in serum-free RPMI with or without 10 µM of 110δ inhibitor (IC87114) in the presence of FITC-conjugated anti-CD19 (HIB19) (BD Biosciences). The cells were incubated for 20 min at 37°C and washed twice. They were fixed and permeabilized according to the BD Phosflow protocol (protocol III). They were then stained and subjected to flow cytometry. The following antibodies were used for staining: Alexa Fluor 647-conjugated anti-phospho AKT (Ser473) (D9E) (Cell Signaling Technology), FITC-conjugated anti-CD19 (BD Biosciences), PE-conjugated anti-CD3 (UCHT1) (BD Biosciences), PE-conjugated anti-CD14 (Mφ97) (BD Biosciences), or FITC-conjugated anti-CD56 (C5.9) (SIGMA-ALDRICH), PE-conjugated CD16 (3G8) (BD Biosciences), and PerCP-Cy 5.5-conjugated anti-CD10 (HI10a) (BD Biosciences). Negative selection of B cells from PBMCs was performed using Pan B-Cell Isolation Kit, human (Miltenyi Biotec Inc., Auburn, CA, USA).

### Statistical Analysis

Receiver operating characteristic (ROC) curves were created with Easy R (EZR) software available online (http://www.jichi.ac.jp/saitama-sct/SaitamaHP.files/statmedEN.html). EZR is statistical software and is based on R and R commander. EZR enables the application of statistical functions (12). Statistical hypotheses were tested using a two-tailed *t*-test. A *p* value < 0.05 was considered significant.

## Results

### Mutation Analysis of Patients With APDS2

**Figure 1:**
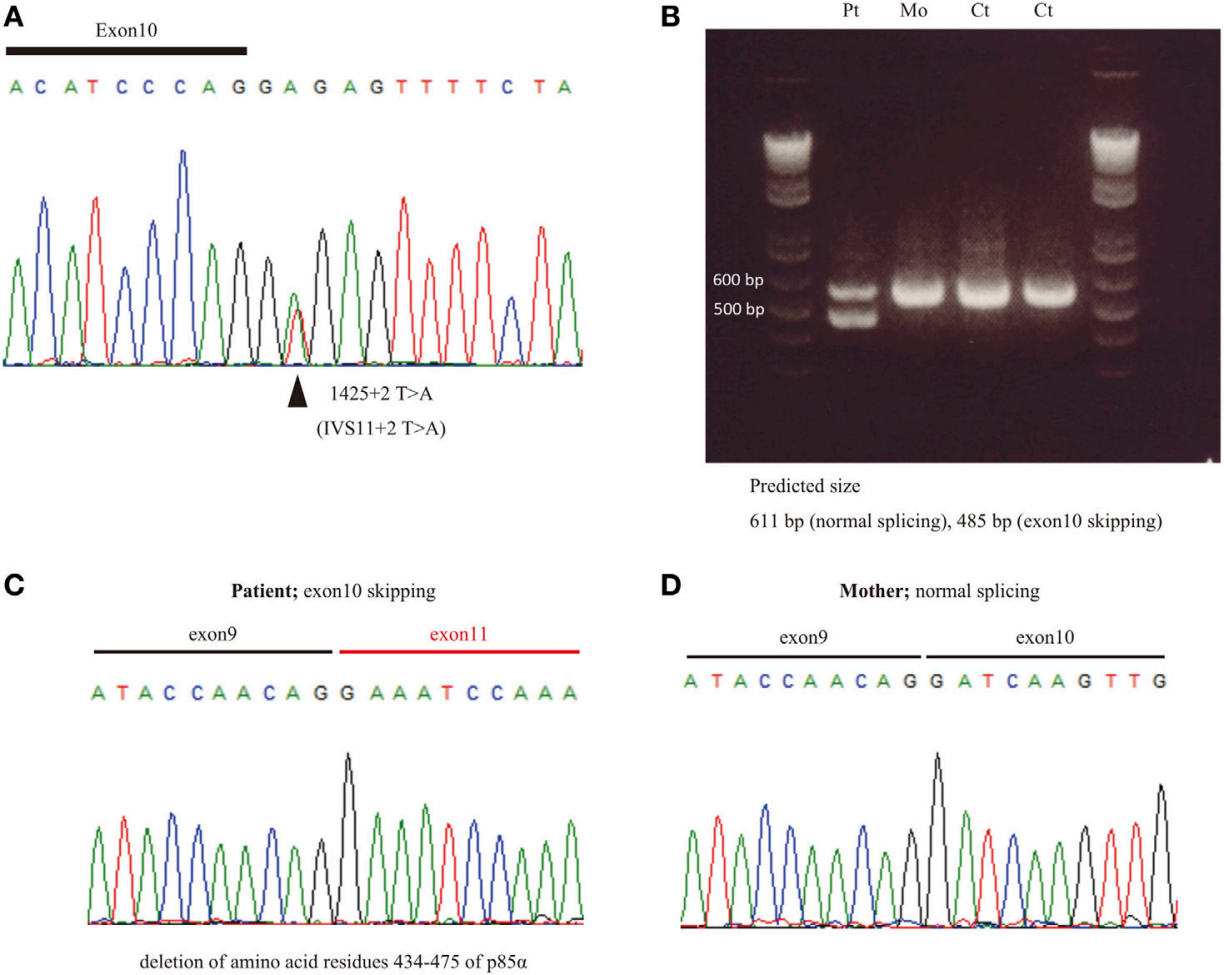
The effect of splice site mutation identified in P1. **(A)** A germline heterozygous mutation, **(B)** 1425 + 2 T > A, in *PIK3R1* was identified by whole exome sequencing and confirmed by Sanger sequencing. **(C)** mRNA was extracted from peripheral blood mononuclear cells from P1 and his mother. Complementary DNA was then synthesized to assess the significance of the nucleotide substitution, 1425 + 2 T > A, on splicing. **(C,D)** The RT-PCR fragment was cloned into a pGEM-T easy vector. Loss of exon 10 in patient **(C)** but not mother **(D)** was revealed by Sanger sequencing.

We found a splice site mutation at the + 2 position following exon 10 (Figure 1A). Amplification of cDNA showed an aberrant, faster migrating band suggesting a deletion (Figure 1B). Sanger sequencing demonstrated a deletion of exon 10 in the patient (Figure 1C) but not in his mother (Figure 1D). Thus, we confirmed that the identified *PIK3R1* mutations in P1, as well as the other two mutations, are pathogenic mutations, leading to the skipping of exon 10 with a deletion of amino acid residues 434–475 of p85α (Figure 1B). The former diagnosis of four patients with APDS2 was CVID (P1), HIGM (P2 and P4), and IgG subclass deficiency (P3). The identified mutations in PIK3R1 were *de novo* in Family B, C, and D, since we found no asymptomatic carrier in a familial study. In order to determine the mechanism of disease in patients with APDS2, we focused on pAKT function associated with PI3K signaling.

### Circulating CD19^+^ B Cells From Patients With APDSs or APDS-L Showing Enhanced pAKT Signaling

**Figure 2:**
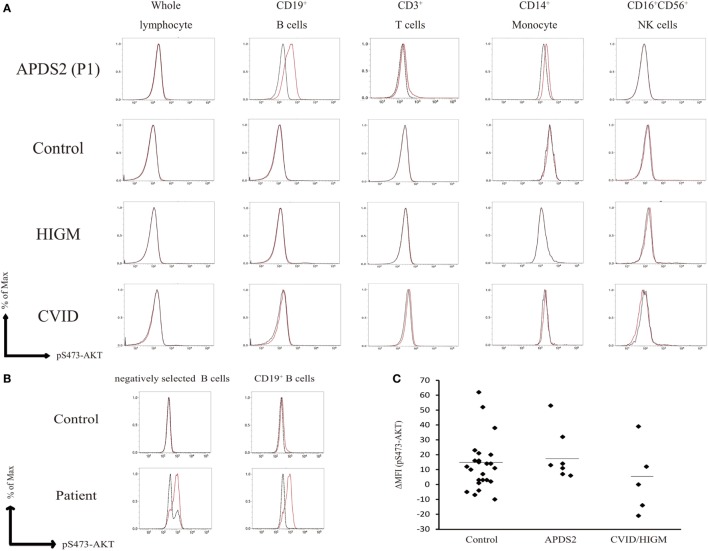
Flow-cytometry-based AKT phosphorylation (pAKT) assay of PMBCs. **(A)** The level of pAKT in the presence or absence of p110δ inhibitor treatment was assessed by flow cytometry in total peripheral blood mononuclear cells (PBMCs), CD3^+^ T cells, CD14^+^ monocytes, and CD16^+^CD56^+^ natural killer cells from activated PI3Kδ syndrome 2 (APDS2) (P1), common variable immunodeficiency (CVID) (P8), or hyper IgM syndrome (HIGM) (P9). The number of events analyzed was >5,000. Red bold line: no treatment and black dotted line: p110δ inhibitor treatment. **(B)** The level of pAKT in negatively selected B cells from PBMCs from P1 and one healthy control was compared with pAKT in CD19^+^ B cells. Red solid line: no treatment and black dotted line: p110δ inhibitor treatment. **(C)** A summary of difference in mean fluorescence intensity of whole lymphocytes derived from 24 adult healthy controls or all of the patients with APDS2 or CVID/HIGM. There is no statistical significant among this three groups (control vs. CVID; *p* = 0.24, APDS2 vs. CVID; *p* = 0.13, and control vs. APDS2; *p* = 0.52).

Enhanced pAKT signaling associated with hyperactive PI3K signaling is a common finding in patients with APDSs (1–4). We first assessed the status of pAKT in fresh (non-cultured) PBMCs from APDS2 patients by flow cytometry. There was no obvious difference in the level of pAKT in CD3^+^ T cells, CD16^+^CD56^+^ natural killer (NK) cells, and CD14^+^ monocytes between APDS2 patients and healthy controls (Figure 2A). In P1’s CD14^+^ monocytes, pAKT was slightly enhanced in the absence of p110δ inhibitor treatment compared with untreated, but this difference was non-significant. In contrast, CD19^+^ B cells from the APDS2 patient (P1) had significantly higher levels of pAKT compared with those from healthy controls. By treating them with a p110δ inhibitor, the enhancement of pAKT observed in CD19^+^ B cells was normalized. In order to exclude the possibility of stimulation of B cells by staining them with anti-CD19 antibody, we confirmed this finding by analyzing circulating B cells separated by negative selection (Figure 2B).

**Figure 3:**
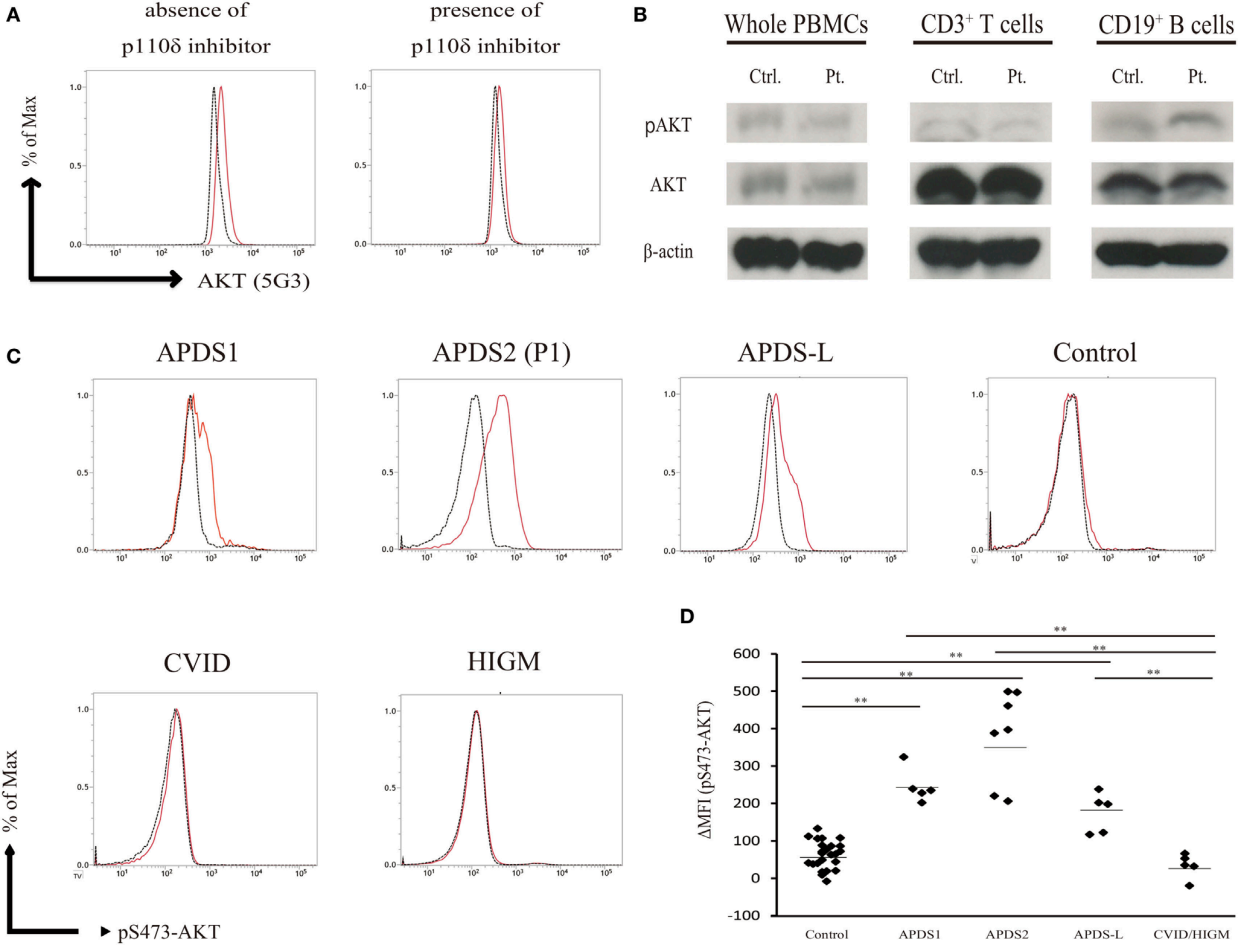
Enhanced AKT phosphorylation (pAKT) in CD19^+^ B cells from patients with activated PI3Kδ syndromes (APDSs) or APDS-like (APDS-L). **(A)** Total AKT expression in CD19^+^ B cells from APDS2 patients (P1) was measured by flow-cytometry. Red bold line: activated PI3Kδ syndrome 2 (APDS2) patient and black dotted line: healthy control. **(B)** The phosphorylation and protein expression of AKT was investigated by immunoblotting. **(C)** pAKT (pS473) in the presence of p110δ inhibitor was analyzed by flow cytometry in CD19^+^ B cells from healthy controls and patients with APDS1, APDS2, or APDS-L, CVID/HIGM. The number of events analyzed was >5,000. **(D)** A summary of difference in mean fluorescence intensity of CD19^+^ B cells derived from healthy controls or patients with APDS1 (four patients), APDS2 (P1, P2, P3, and P4), APDS-L (four patients), or CVID/HIGM (P5, P6, P7, P8, and P9). *p < 0.05 and **p < 0.01.

We measured the mean fluorescence intensity (MFI) of pAKT in the presence or absence of p110δ inhibitor treatment by flow cytometry. We then evaluated enhanced pAKT signaling by calculating the difference in MFI (ΔMFI) of pAKT in CD19^+^ B cells as the difference between MFI (the absence of p110δ inhibitor) and MFI (the presence of p110δ inhibitor). CD19^+^ B cells from APDS2 patients had significantly higher ΔMFI of pAKT than those from healthy controls or CVID/HIGM patients, although ΔMFI of pAKT in whole lymphocytes was almost at the same levels among all individuals (Figures 2C and 3D). The protein expression of total AKT in CD19^+^ B cells from an APDS2 patient (P1) was equivalent to that in healthy controls (Figure 3A). The result was consistent with the previous studies showing the normal AKT protein expression in APDSs and APDS-L patients (1–4, 6). The enhancement of pAKT in CD19^+^ B cells was confirmed by immunoblotting (Figure 3B; Figure S2 in Supplementary Material). In contrast, there was no difference in the level of pAKT in CD3^+^ T cells (Figure 3B; Figure S2 in Supplementary Material).

We next tested the hypothesis that enhanced pAKT signaling in CD19^+^ B cells is a common finding of patients with hyperactive PI3K signaling. We analyzed PBMCs from patients with APDS1 or APDS-L carrying a heterozygous mutation in *PIK3CD* or *PTEN*, respectively. As expected, CD19^+^ B cells from APDS1 and APDS-L showed significantly higher levels of pAKT compared with healthy controls and CVID/HIGM patients (Figure 3C). As above, the enhancement of pAKT observed in these patients was normalized by treatment with a p110δ inhibitor. Similar to the results obtained from patients with APDS2, the level of pAKT was normal in CD3^+^ T cells, CD16^+^CD56^+^ NK cells, and CD14^+^ monocytes from patients with APDS1 or APDS-L (data not shown). However, CD19^+^ B cells and other cell populations from patients with CVID and HIGM had normal levels of pAKT expression (Figure 2A). Curiously, CD19^+^ B cells from APDS2 patients had the highest ΔMFI of pAKT among all APDSs patients (Figure 3D), followed by elevated ΔMFI of pAKT in APDS-L patients observed as significantly higher than healthy controls and CVID/HIGM patients (Figure 3D). In addition, higher levels of pAKT were also observed in CD19^+^ B cells from cryopreserved PBMCs (Figures S3A,B in Supplementary Material). Therefore, the enhancement of pAKT in CD19^+^ B cells was considered to be a specific finding among patients with APDSs and APDS-L.

### Enhanced pAKT Signaling in Activated T Cells From APDS2 Patients

**Figure 4:**
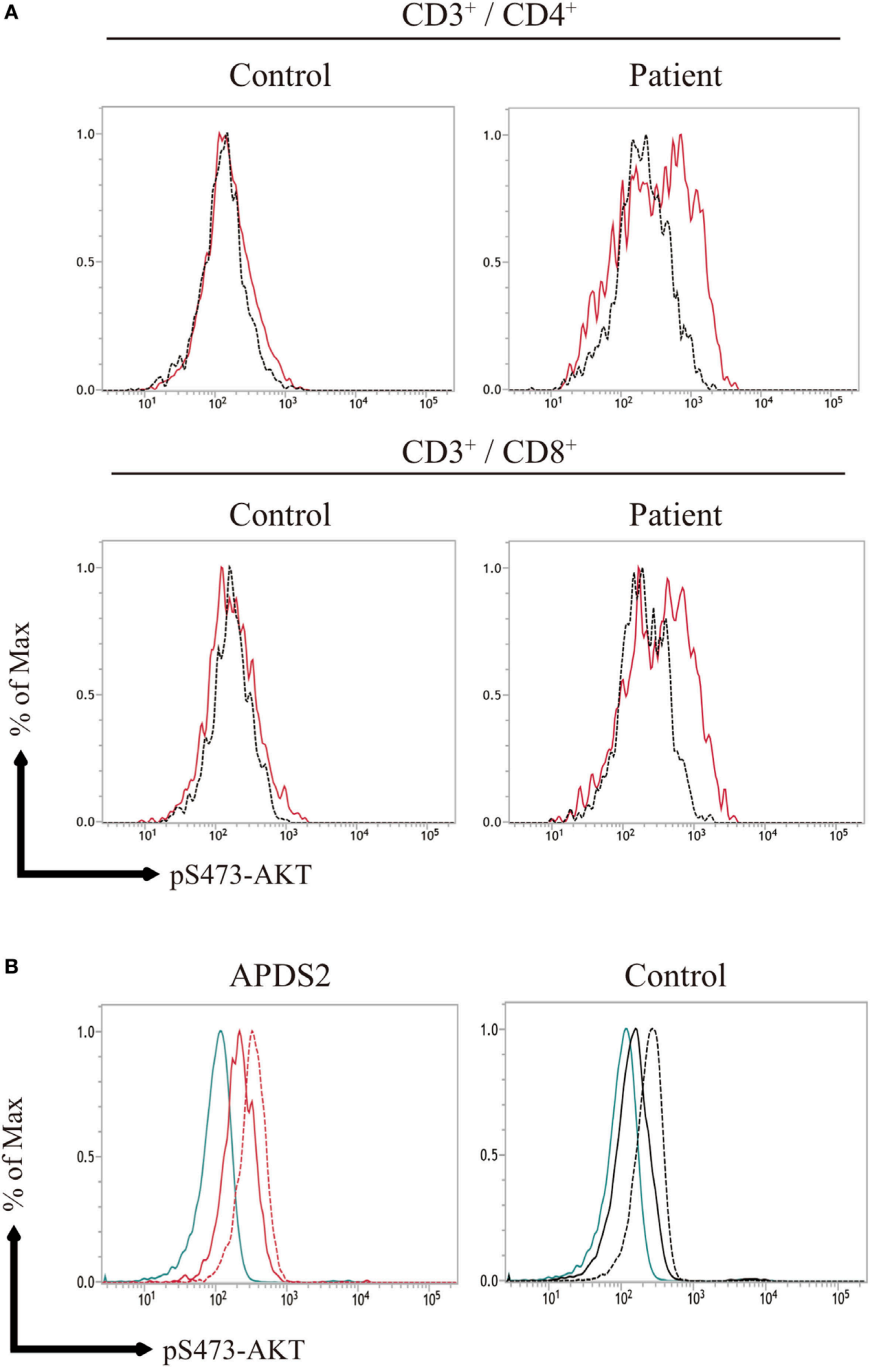
**(A)** AKT phosphorylation (pAKT) expression in activated T cells, **(B)** pAKT expression CD19^+^ B cells stimulated with CD40L and IL-4. **(A)** The level of pAKT in T cells was assessed by flow cytometry in activated PI3Kδ syndrome 2 patient (P1) and healthy controls (representative result from two controls is shown). The total number of events analyzed was 2,000. Red bold line: no treatment and black dotted line: p110δ inhibitor treatment. **(B)** pAKT expression in CD19^+^ B cells from (P1) and healthy control following stimulation with CD40L and IL-4. Blue line: isotype control, black and red solid line: no treatment, and black and red dot line: CD40L and IL-4 treatment.

The previous study showed the enhancement of pAKT signaling in activated T cells from APDSs (1–4). We next investigated pAKT levels in activated T cells derived from PBMCs by flow cytometry. CD3^+^CD4^+^ and CD3^+^CD8^+^-activated T cells from P1 showed enhanced pAKT compared with those from healthy controls (Figure 4A). The enhancement of pAKT observed in P1 was normalized by treating cells with a p110δ inhibitor. The result was consistent with previous studies that investigated activated T cells from patients with APDSs (1–4). Thus, we confirmed the enhancement of pAKT in activated T cells from APDS2 patients by flow cytometry. Next, we stimulated B cells with CD40L and IL-4 and investigated the level of pAKT. Following stimulation with CD40L and IL-4, we observed the enhancement of pAKT in CD19^+^ B cells from APDS2 patient and healthy control (Figure 4B). However, this difference became less striking after CD40L and IL-4 stimulation.

### The Enhancement of pAKT in Patients With APDSs Pronounced in CD10^+^CD19^+^ Immature B Cells

**Figure 5:**
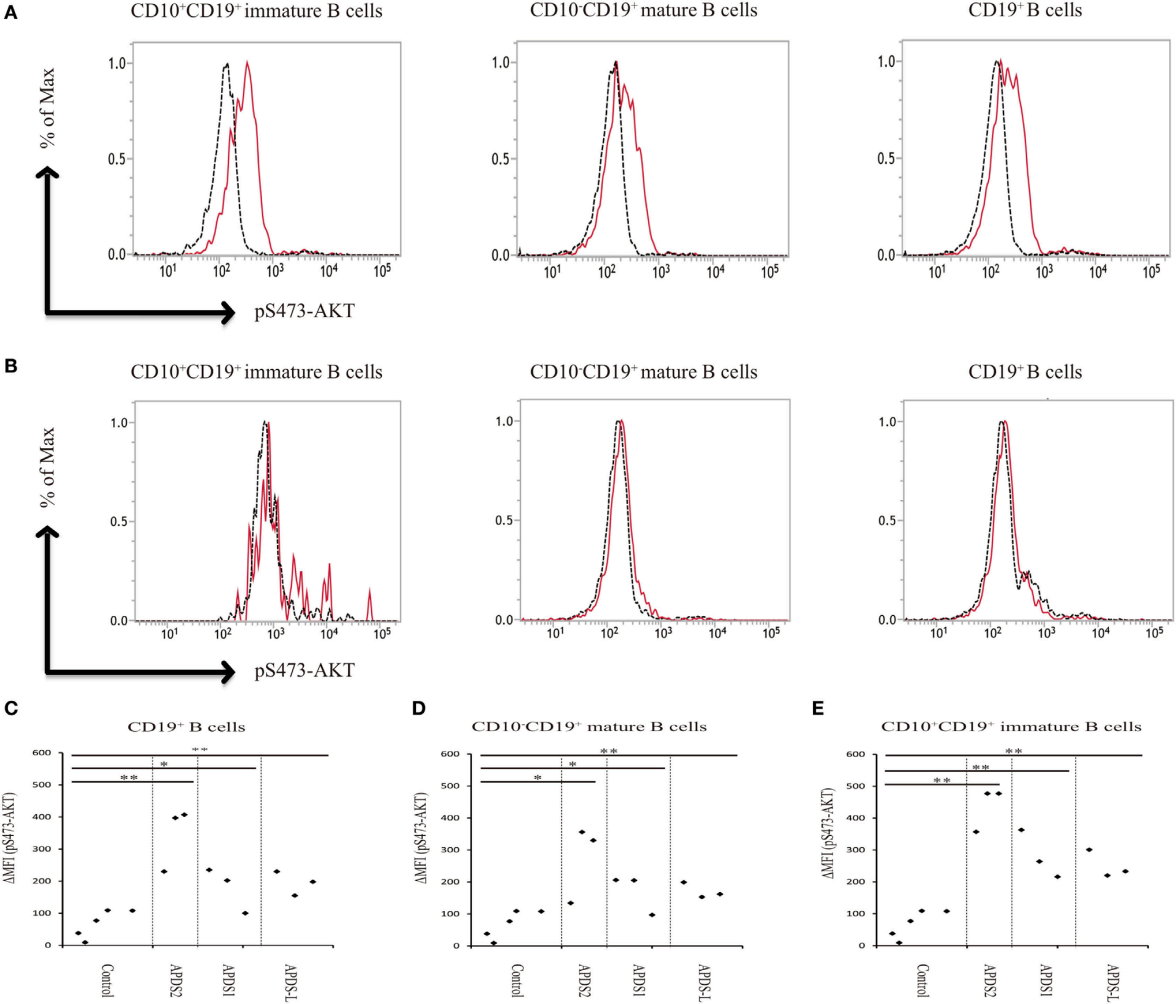
Enhanced AKT phosphorylation (pAKT) in activated PI3Kδ syndrome 2 (APDS2) patients was pronounced in a flow-cytometry assay of CD10^+^CD19^+^ B cells. **(A,B)** The enhancement of pAKT observed in CD19^+^ B cells was further investigated by separating them into CD10^+^CD19^+^ immature B cells and CD10^−^CD19^+^ mature B cells from P2 (representative histogram of three experiments) **(A)** and healthy controls **(B)**. The number of events analyzed was >5,000. As an exception, CD10^+^CD19^+^ immature B cells analyzed in healthy control were 800 events. Red solid line: no treatment and black dotted line: p110δ inhibitor treatment. **(C,D,E)** A summary of difference in mean fluorescence intensity of CD19^+^ B cells **(C)**, CD10^−^CD19^+^ mature B cells **(D)**, and CD10^+^CD19^+^ immature B cells **(E)** from healthy controls or patients with APDS1 (three patients), APDS2 (P1, P2, and P4), or APDS-like (APDS-L) (three patients). *p < 0.05 and **p < 0.01.

Phosphoinositide 3-kinase signaling has important roles in differentiation, development, and functions in several distinct stages of B and T cells (7, 8). Patients with APDS1, APDS2, or APDS-L had increased numbers of transitional B cells in the peripheral blood, possibly reflecting the pivotal role of PI3K signaling in the differentiation of B cells (1–5). In our study, APDS2 patients had increased numbers of transitional B cells in peripheral blood consistent with previous studies (Figure S4 in Supplementary Material). We investigated pAKT levels in CD19^+^ B cells by dividing them into three developmental stages: (i) total CD19^+^ B cells, (ii) CD10^−^CD19^+^ mature B cells, and (iii) CD10^+^CD19^+^ immature B cells (corresponding to transitional B cells) by flow cytometry (Figure S5 in Supplementary Material). CD19^+^ B cells at all three developmental stages from patients with APDS1, APDS2, or APDS-L had higher levels of pAKT compared with healthy controls. Surprisingly, the enhancement of pAKT was most pronounced in CD10^+^CD19^+^ immature B-cell populations from APDS1, APDS2, or APDS-L patients (Figure 5). This finding was confirmed by CD10^+^ negatively selected B cells, controlling for the potential B-cell activation by anti-CD19 antibody staining (Figure S6 in Supplementary Material).

### Establishment of a Flow-Cytometry-Based Rapid Discrimination Assay Based on the Enhancement of PI3K Signaling

We found that CD19^+^ B cells from APDSs or APDS-L patients had significantly higher levels of pAKT than healthy controls and patients with CVID or HIGM. The higher level of pAKT observed in these patients was normalized by treating the cells with a p110δ inhibitor. This discovery led us to the idea that the detection of enhanced pAKT signaling in CD19^+^ B cells is a useful diagnostic tool for the rapid discrimination study of suspected APDS patients. CD19^+^ B cells from patients with APDS2 had the highest ΔMFI of pAKT among all APDSs or APDS-L patients. In contrast, elevated ΔMFI of pAKT in CD19^+^ B cells was modest in APDS-L patients (Figure 3D). We next analyzed the ΔMFI of pAKT in CD10^−^CD19^+^ and CD10^+^CD19^+^ B cells. As expected, the ΔMFI of pAKT was high in both B-cell populations from APDSs or APDS-L patients when compared with those from healthy controls (Figures 5C–E). The high ΔMFI of pAKT was emphasized in the analysis of CD10^+^CD19^+^ immature B cells (Figure 5E). Moreover, if we focused on CD10^+^CD19^+^ immature B cells, there was no overlap in the value of ΔMFI between APDSs and APDS-L patients and the other populations including healthy controls and CVID/HIGM patients. This finding strongly suggests that the flow-cytometry-based assay to measure the ΔMFI of pAKT can be used as a discrimination assay to detect the enhancement of PI3K activity in patients with APDSs and APDS-L (flow chart is shown in Figure S7 in Supplementary Material).

### Cutoff Value of ΔMFI of pAKT Segregating APDSs and APDS-L Patients

We created an ROC curve based on the results of the ΔMFI of pAKT obtained from the analysis of CD19^+^ B cells. We used EZR for statistical analysis and set up the cutoff value of ΔMFI of pAKT as 117. This cutoff value allows the segregation of APDSs and APDS-L patients from healthy controls or CVID patients with 100% sensitivity and 96.0% specificity (Figure S8 in Supplementary Material). The area under the curve was 0.996 (95% confidence interval 0.986−1.000) (12).

## Discussion

Here, we investigated four Japanese cases with APDS2 carrying a heterozygous mutation in *PIK3R1*. Elkaim et al. recently summarized the clinical and immunological aspects of 36 genetically diagnosed APDS2 patients and revealed that recurrent upper respiratory tract infections (100%), pneumonitis (71%), and chronic lymphoproliferation (89%) were the most common clinical features (11). Malignant diseases were identified in 28% of patients, most of them were B-cell lymphomas. Laboratory findings showed that patients with APDS2 had decreased serum IgA and IgG levels (87%), increased IgM levels (58%), B-cell lymphopenia (88%), and an increased frequency of transitional B cells (93%) (11). All four patients developed recurrent upper or lower respiratory tract infections and showed decreased serum IgG, which required intravenous immunoglobulin replacement therapy. Elevated serum IgM was observed in two patients, and one patient developed malignant lymphoma. Therefore, the four Japanese patients in the current study were considered to be typical cases of APDS2.

The former diagnoses of the four patients in the current study were HIGM (P2 and P4), CVID (P1), and IgG subclass deficiency (P3). Based on the phenotypic diversity and similarity in clinical and laboratory findings, considerable numbers of patients with APDS2, as well as patients with APDS1 (13), have been historically diagnosed as HIGM or CVID. From the first identification of APDS1 associated with hyperactive PI3K signaling, a definitive diagnosis of APDSs is performed by the identification of mutations in *PIK3CD* or *PIK3R1*. In addition to these genetic tests, the detection of enhanced pAKT signaling in T-cell blasts by functional assays has been used to confirm hyperactive PI3K signaling. However, this assay method is not suitable for rapid diagnosis, because it requires the cultivation of T cells. In the current study, we observed significantly higher levels of pAKT in CD19^+^ B cells, but not CD3^+^ T cells, CD16^+^CD56^+^ NK cells, or CD14^+^ monocytes isolated from fresh PBMCs from APDSs and APDS-L patients. The enhancement of pAKT in CD19^+^ B cells was pronounced in CD10^+^CD19^+^ immature B cells. Moreover, when we focused on this CD10^+^CD19^+^ B-cell population, there was no overlap in the value of ΔMFI of pAKT between APDSs or APDS-L patients and the other populations, including healthy controls and patients with CVID or HIGM. We also made a similar observation using cryopreserved PBMCs from patients with APDSs or APDS-L. This finding allowed us to perform the rapid detection of hyperactive PI3K signaling without culturing patient cells. Although further studies are required to optimize and evaluate this flow-cytometry-based assay system, this assay system has a potential to enable a rapid diagnosis of APDSs and APDS-L

In this study, the enhanced ΔMFI of pAKT in CD19^+^ B cells was a common finding among patients with APDSs or APDS-L. This observation was further enhanced if we focused on CD10^+^CD19^+^ immature B cells. These observations may reflect the importance of PI3K signaling in class-switch recombination in B cells (9, 14), and be related to abnormalities in the developmental stages of B cells, such as abnormalities of the germinal center structure (11, 15–17), increased circulating transitional B cells, and decreased class switching B cells in patients with APDSs (1–4). Curiously, the ΔMFI of pAKT was the highest in CD19^+^ B cells from APDS2 patients and was the lowest in CD19^+^ B cells from APDS-L patients. It is interesting to speculate possible molecular mechanism underlie this observation. The p85α is known to enhance enzymatic activity of PTEN (18). Although PTEN protein expression is normal in APDS2 patients (2), its enzymatic activity might be affected by functional impairment of p85α. Therefore, impairment of p85α may enhance pAKT by losing its inhibitory role of p110δ and its enhancing effect against PTEN enzymatic activity.

Relatively mild enhancement of pAKT in APDS-L patients might explain the clinical observation that only a part of patients with PHTS, caused by heterozygous mutations in PTEN, present with antibody deficiency (5). Indeed, the clinical penetrance of APDS-L is not high in patients with PHTS (19). There are considerable overlaps in the clinical manifestations between APDS1 and APDS2 (Table 1) (1–4, 11, 20); however, there are also some differences in these two disorders. Indeed, patients with APDS2 have a higher susceptibility to lymphoma than patients with APDS1 (11, 20, 21) (Okano et al., under revision). The clinical penetrance of APDS1 is quite high, but not complete, possibly explaining the existence of asymptomatic carriers or cases with mild symptoms that only show recurrent respiratory infections and diagnosed as APDS1 by familial studies that identified a proband case (20). However, to date no asymptomatic carriers have been reported in patients with APDS2. Although we require large cohort studies to make strong conclusions, these clinical observations might be partially explained by the difference in elevated ΔMFI of pAKT. Further studies are necessary to understand the role of elevated ΔMFI of pAKT in CD19^+^ B cells on the immunological manifestations among patients with APDSs and APDS-L. The selective effect of pAKT in B cells (transitional B cells in particular), which may provide a detailed pathological mechanism of APDSs and APDS-L, remains to be explained.

**Table 1:** Clinical features of patients with APDSs, APDS-L, or CVID.

Clinical feature	APDS1 (%)	APDS2 (%)	APDS-L (%)	CVID (%)
Pneumonia	85	71	50	32–77
Lymphoproliferation	75	89	44	N.D.
Splenomegaly	58	43	N.D.	15–30
Enteropathy	25	24	N.D.	9
Granuloma	0	N.D.	N.D.	8–9
Meningitis/encephalitis	1.9	N.D.	N.D	3–4
Autoimmunity	42	17	N.D.	22–29
Malignancy	13	28	22	3–8
Neurodevelopmental delay	19	31	50	N.D.
IVIG therapy	77	89	19	80
Reference	(20)	(11)	(5, 6)	(20)

Recently, molecular targeting therapy using an mTOR inhibitor was also effective for the treatment of lymphoproliferation in patients with APDSs (1, 3, 11). Therefore, the prompt and appropriate diagnosis of APDSs definitely benefits patients by providing a therapeutic choice of target therapy. The flow-cytometry-based rapid assay of PI3K activity described here has the potential to provide a rapid diagnosis of APDSs and APDS-L.

## Concluding Remarks

The flow-cytometry-based rapid assay of PI3K activity described here provides a rapid discrimination assay of identified mutations in *PIK3CD, PIK3R1*, and *PTEN*, and might also be a potential diagnostic tool for patients with APDSs or APDS-L.

## Ethics Statement

We obtained written informed consent for genomic analysis and blood-sample-based functional studies of the patients, parents, and siblings in accordance with the Declaration of Helsinki. The genetic analysis and blood-sample-based functional studies were approved by the Institutional Review Board of Hiroshima University.

## Author Contributions

Patient workup: TA, SO, KM-S, YT, YI, AS, KH, TW, KI, TM, SN, and MK. Flow-cytometry analysis: TA and MT. Drafting the manuscript: TA, SO, and KM. Final approval of the version to be published: TA, SO, MT, T-WY, KM-S, YT, YI, AS, KH, TW, KI, OO, TM, SN, and MK. Agreement to be accountable for all aspects of the work: TA, SO, MT, T-WY, KM-S, YT, YI, AS, KH, TW, KI, OO, TM, SN, and MK.

## Conflict of Interest Statement

The authors declare that the research was conducted in the absence of any commercial or financial relationships that could be construed as a potential conflict of interest.

## Abbreviations

APDS, activated PI3Kδ syndrome; APDS1, activated PI3Kδ syndrome 1; APDS2, activated PI3Kδ syndrome 2; APDS-L, activated PI3Kδ syndrome like immunodeficiency; APDSs, activated PI3Kδ syndrome 1 and activated PI3Kδ syndrome 2; AUC, area under the curve; cDNA, complementary DNA; CI, confidence interval; CMV, cytomegalovirus; CVID, common variable immunodeficiency; ΔMFI, difference in mean fluorescence intensity; EZR, Easy R; EBV, Epstein Barr virus; GOF, gain of function; HIGM, hyper IgM syndrome; IVIG, intravenous immunoglobulin; LOF, loss of function; MFI, mean fluorescence intensity; mTOR, mechanistic target of rapamycin; NK, natural killer; PBMCs, peripheral blood mononuclear cells; PCR, polymerase chain reaction; PI3K, phosphoinositide 3-kinase; PID, primary immunodeficiency syndrome; PIP2, phosphatidylinositol 3, 4- triphosphate; PIP3, phosphatidylinositol 3, 4, 5- triphosphate; PHTS, PTEN Hamartoma Tumor Syndromes; PTEN, phosphatase and tensin homolog; ROC, receiver operating characteristic; Ser473, Serine473; pAKT, AKT phosphorylation.

## References

[1] Angulo I Vadas O Garcon F Banham-Hall E Plagnol V Leahy TR Phosphoinositide 3-kinase delta gene mutation predisposes to respiratory infection and airway damage. Science (2013) 342(6160):866–71. 10.1126/science.1243292 24136356 PMC3930011

[2] Deau MC Heurtier L Frange P Suarez F Bole-Feysot C Nitschke P A human immunodeficiency caused by mutations in the PIK3R1 gene. J Clin Invest (2014) 124(9):3923–8. 10.1172/JCI75746 25133428 PMC4153704

[3] Lucas CL Kuehn HS Zhao F Niemela JE Deenick EK Palendira U Dominant-activating germline mutations in the gene encoding the PI(3)K catalytic subunit p110delta result in T cell senescence and human immunodeficiency. Nat Immunol (2014) 15(1):88–97. 10.1038/ni.2771 24165795 PMC4209962

[4] Lucas CL Zhang Y Venida A Wang Y Hughes J McElwee J Heterozygous splice mutation in PIK3R1 causes human immunodeficiency with lymphoproliferation due to dominant activation of PI3K. J Exp Med (2014) 211(13):2537–47. 10.1084/jem.20141759 25488983 PMC4267241

[5] Driessen GJ IJspeert H Wentink M Yntema HG van Hagen PM van Strien A Increased PI3K/Akt activity and deregulated humoral immune response in human PTEN deficiency. J Allergy Clin Immunol (2016) 138(6):1744–7.e5. 10.1016/j.jaci.2016.07.010 27531073

[6] Tsujita Y Mitsui-Sekinaka K Imai K Yeh TW Mitsuiki N Asano T Phosphatase and tensin homolog (PTEN) mutation can cause activated phosphatidylinositol 3-kinase delta syndrome-like immunodeficiency. J Allergy Clin Immunol (2016) 138(6):1672–80.e10. 10.1016/j.jaci.2016.03.055 27426521

[7] Koyasu S. The role of PI3K in immune cells. Nat Immunol (2003) 4(4):313–9. 10.1038/ni0403-313 12660731

[8] Okkenhaug K Vanhaesebroeck B PI3K in lymphocyte development, differentiation and activation. Nat Rev Immunol (2003) 3(4):317–30. 10.1038/nri1056 12669022

[9] Omori SA Cato MH Anzelon-Mills A Puri KD Shapiro-Shelef M Calame K Regulation of class-switch recombination and plasma cell differentiation by phosphatidylinositol 3-kinase signaling. Immunity (2006) 25(4):545–57. 10.1016/j.immuni.2006.08.015 17000121

[10] Maehama T Dixon JE. The tumor suppressor, PTEN/MMAC1, dephosphorylates the lipid second messenger, phosphatidylinositol 3,4,5-trisphosphate. J Biol Chem (1998) 273(22):13375–8. 10.1074/jbc.273.22.13375 9593664

[11] Elkaim E Neven B Bruneau J Mitsui-Sekinaka K Stanislas A Heurtier L Clinical and immunologic phenotype associated with activated phosphoinositide 3-kinase delta syndrome 2: a cohort study. J Allergy Clin Immunol (2016) 138(1):210–8.e9. 10.1016/j.jaci.2016.03.022 27221134

[12] Kanda Y. Investigation of the freely available easy-to-use software ’EZR’ for medical statistics. Bone Marrow Transplant (2013) 48(3):452–8. 10.1038/bmt.2012.244 23208313 PMC3590441

[13] Elgizouli M Lowe DM Speckmann C Schubert D Hulsdunker J Eskandarian Z Activating PI3Kdelta mutations in a cohort of 669 patients with primary immunodeficiency. Clin Exp Immunol (2016) 183(2):221–9. 10.1111/cei.12706 26437962 PMC4711166

[14] Dengler HS Baracho GV Omori SA Bruckner S Arden KC Castrillon DH Distinct functions for the transcription factor Foxo1 at various stages of B cell differentiation. Nat Immunol (2008) 9(12):1388–98. 10.1038/ni.1667 18978794 PMC2679692

[15] Lougaris V Faletra F Lanzi G Vozzi D Marcuzzi A Valencic E Altered germinal center reaction and abnormal B cell peripheral maturation in PI3KR1-mutated patients presenting with HIGM-like phenotype. Clin Immunol (2015) 159(1):33–6. 10.1016/j.clim.2015.04.014 25939554

[16] Sander S Chu VT Yasuda T Franklin A Graf R Calado DP PI3 kinase and FOXO1 transcription factor activity differentially control B cells in the germinal center light and dark zones. Immunity (2015) 43(6):1075–86. 10.1016/j.immuni.2015.10.021 26620760

[17] Di Fonte R Baronio M Plebani A Lougaris V Fousteri G Reduced germinal center follicular helper T cells but normal follicular regulatory T cells in the tonsils of a patient with a mutation in the PI3KR1 gene. Clin Immunol (2016) 164:43–4. 10.1016/j.clim.2016.01.016 26827886

[18] Chagpar RB Links PH Pastor MC Furber LA Hawrysh AD Chamberlain MD Direct positive regulation of PTEN by the p85 subunit of phosphatidylinositol 3-kinase. Proc Natl Acad Sci U S A (2010) 107(12):5471–6. 10.1073/pnas.0908899107 20212113 PMC2851819

[19] Chen HH Handel N Ngeow J Muller J Huhn M Yang HT Immune dysregulation in patients with PTEN hamartoma tumor syndrome: analysis of FOXP3 regulatory T cells. J Allergy Clin Immunol (2016) 139(2):607–20.e15. 10.1016/j.jaci.2016.03.059 27477328 PMC5292998

[20] Coulter TI Chandra A Bacon CM Babar J Curtis J Screaton N Clinical spectrum and features of activated phosphoinositide 3-kinase delta syndrome: a large patient cohort study. J Allergy Clin Immunol (2016) 139(2):597–606.e4. 10.1016/j.jaci.2016.06.021 27555459 PMC5292996

[21] Kracker S Curtis J Ibrahim MA Sediva A Salisbury J Campr V Occurrence of B-cell lymphomas in patients with activated phosphoinositide 3-kinase delta syndrome. J Allergy Clin Immunol (2014) 134(1):233–6. 10.1016/j.jaci.2014.02.020 24698326 PMC4671279

