# Supplementary material for: Enhanced AKT Phosphorylation of Circulating B Cells in Patients With Activated PI3Kδ Syndrome
Source: Front Immunol. 2018 Apr 5;9:568. doi: 10.3389/fimmu.2018.00568 (PMC5895775; doi:10.3389/fimmu.2018.00568)
Supplement: Supplementary file 1 [file data_sheet_1.DOCX]

Supplementary Material

**Enhanced AKT phosphorylation of circulating B cells in patients with activated PI3Kδ syndrome**

**Takaki Asano^1^, Satoshi Okada^1*^, Miyuki Tsumura^1^, Tzu-Wen Yeh^2^, Kanako Mitsui-Sekinaka^4^, Yuki Tsujita^4^, Youjiro Ichinose^5^, Akira Shimada^6^, Kunio Hashimoto^7^, Taizo Wada^8^, Kohsuke Imai^3^, Osamu Ohara^9^, Tomohiro Morio^2^, Shigeaki Nonoyama^4^, Masao Kobayashi^1^**

*** Correspondence: Satoshi Okada**: [saok969@gmail.com](mailto:saok969@gmail.com)


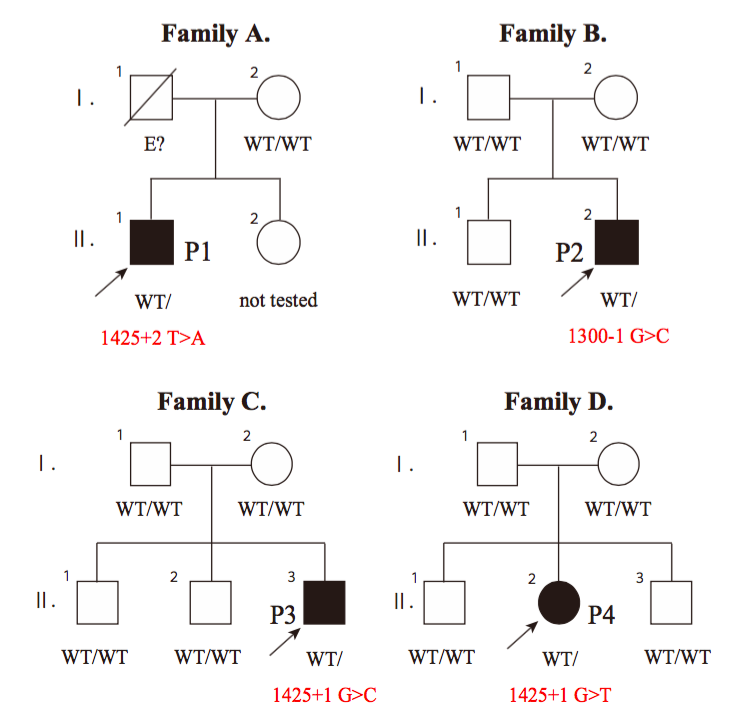


**SUPPLEMENTAL FIGURE 1**

**Family trees**

Family tree of four Japanese patients with APDS2. The detailed clinical description is available in Supplemental material and methods. The mutation was *de novo* in Family B, C and D. No asymptomatic carrier was identified in the familial study. E?; not tested.

**
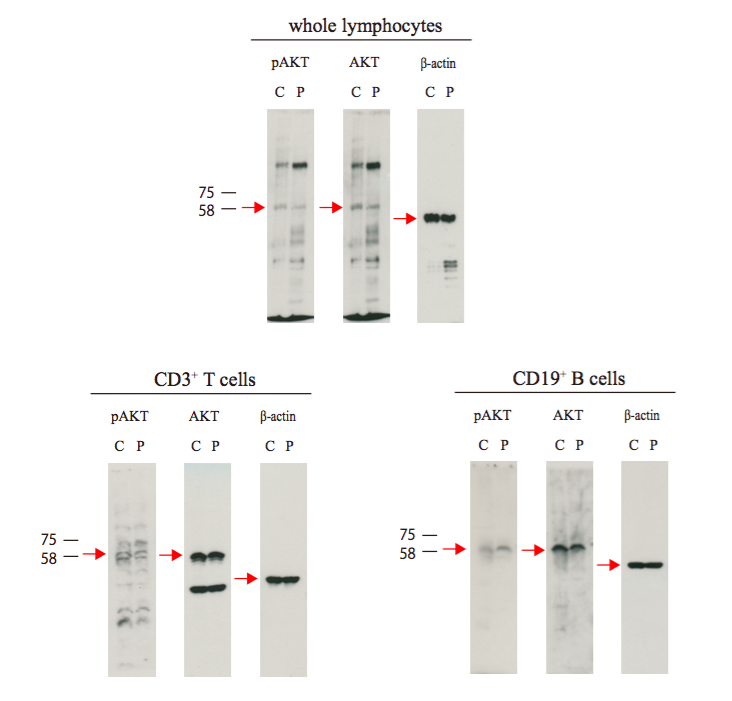
**

**SUPPLEMENTAL FIGURE 2**

**The original image of immunoblot analyzing pAKT, AKT and b-actin**

The original image of immunoblot of Figure 3B were shown. The red arrow indicates the bands of pAKT (60 kb), AKT (60 kb) and β-actin (42 kb). Healthy Control: C, APDS2 patient: P.


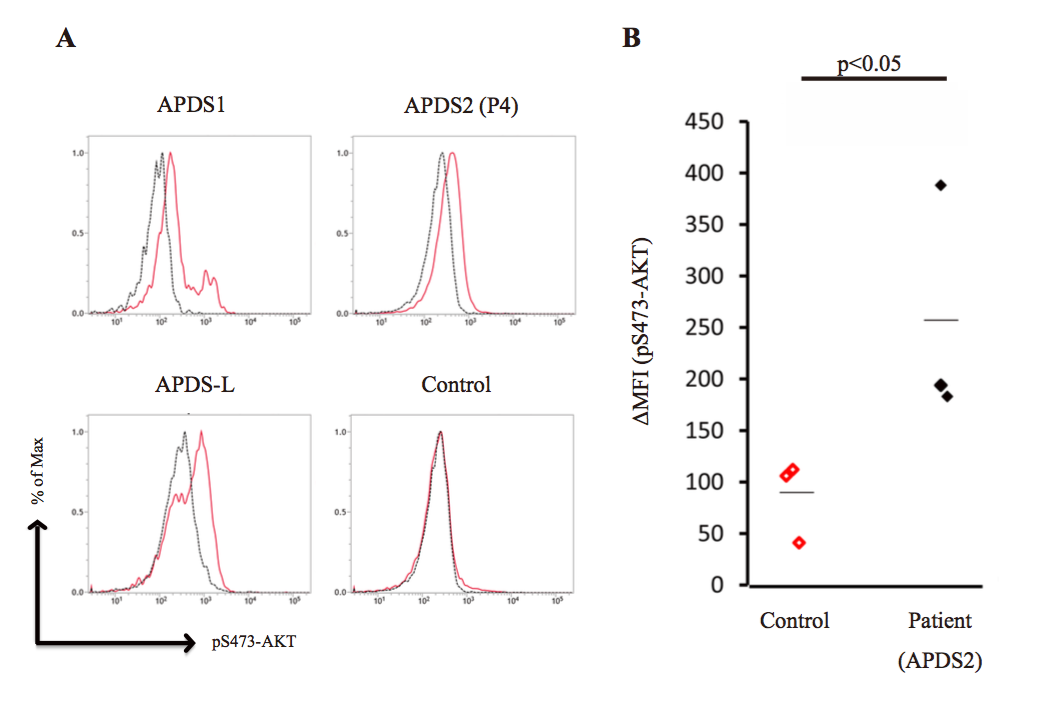


**SUPPLEMENTAL FIGURE 3**

**pAKT analysis in cryopreserved CD19^+^ B cells**

(**A**) Cryopreserved PBMCs from APDS1, APDS2 and APDS-L patients and a healthy control were assessed by flow cytometry. CD19^+^ B cells from APDS1, APDS2 (P4), and APDS-L patients, but not those from a healthy control, showed the enhancement of pAKT that was normalized in the presence of p110δ inhibitor treatment. Red solid line: no treatment, black dotted line; p110δ inhibitor treatment. (**B**) A summary of ΔMFI of pAKT in cryopreserved CD19^+^ B cells from healthy controls and a patient with APDS2 (P1, P2, and P4). The ΔMFI was significantly higher in cryopreserved CD19^+^ B cells from APDS2 patients than those from healthy controls (p<0.05).

**
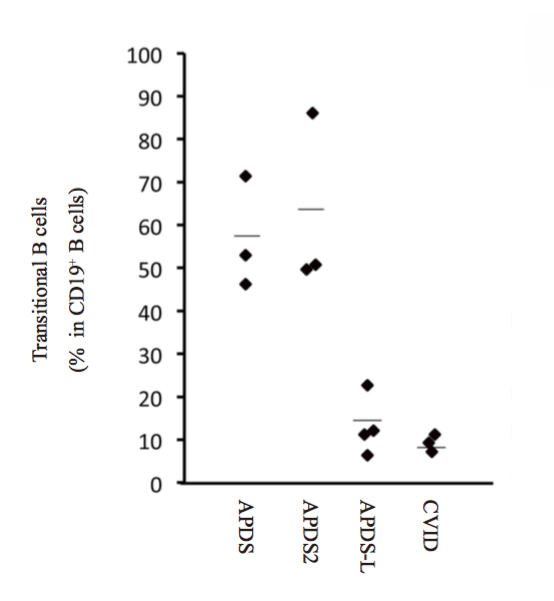
**

**SUPPLEMENTAL FIGURE 4**

**The percentage of transitional B cells in CD19^+^ B cells**

The percentage of transitional B cells in CD19^+^ B cells in patients with APDS1, APDS2 (P2, P3, and P4), APDS-L and CVID (P6, P7, and P8) are shown. Similar to previous studies, the percentage of transitional B cells was higher in APDS1/APDS2 patients than CVID patients (p<0.01). The percentage of transitional B cells was not investigated in a patient with HIGM (P9). There is no statistical significant in this two groups (APDS-L vs CVID; p= 0.33 and APDS vs APDS2; p=0.67). There is statistical significant among this four groups (APDS vs APDS-L, APDS vs CVID, APDS2 vs APDS-L, and APDS2 vs CVID; p<0.01)


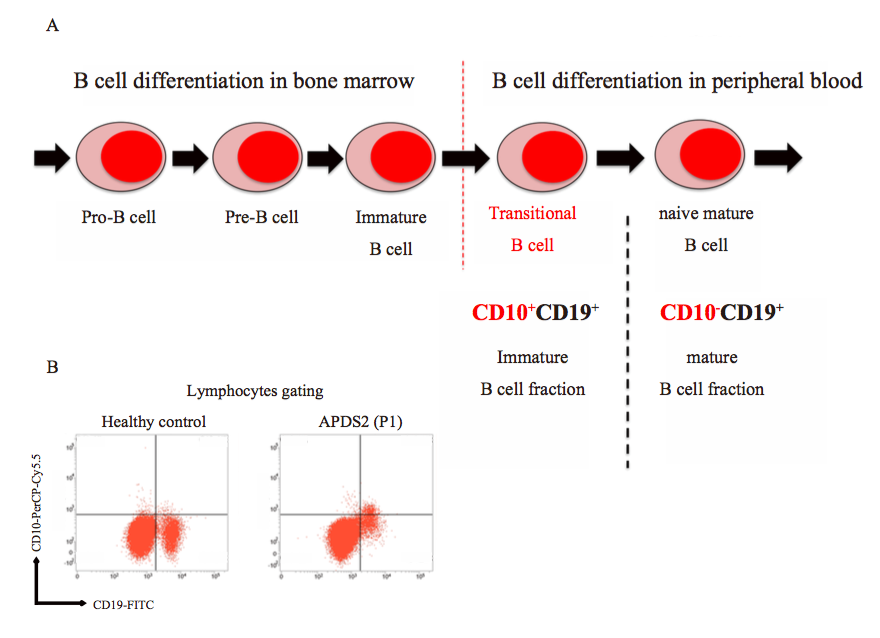


**SUPPLEMENTAL FIGURE 5**

**Scheme of B cell differentiation and maturation**

(A) The process of B cell differentiation and maturation is shown. CD19^+^ B cells are divided into CD10^+^ CD19^+^ immature B and CD10^-^ CD19^+^ mature B cells and subjected to the analysis of pAKT. (B) The representative quadrant plot of CD19-FITC and CD10-Cy5.5 staining gated on lymphocytes. After lymphocytes gating, they are divided into three developmental stages by flow cytometry as follows; i) CD19^+^ B cells (upper and lower right quadrant), ii) CD10^-^CD19^+^ mature B cells (lower right quadrant), and iii) CD10^+^CD19^+^ immature B cells (upper right quadrant) which corresponds to transitional B cells.


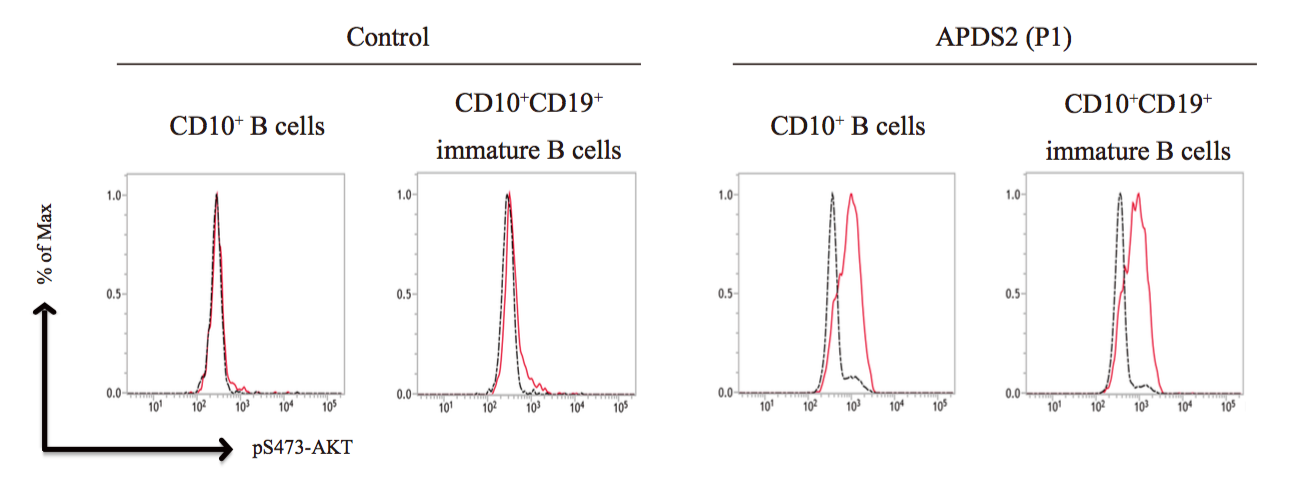


**SUPPLEMENTAL FIGURE 6**

**The impact on pAKT in B cells by FCM antibodies**

The level of pAKT expression in CD10^+^ B cells was assessed in negatively selected B cells purified from PBMCs. There were no differences in pAKT activity between CD10^+^CD19^+^ B cells and CD10^+^ B cells. Red solid line: no treatment, black dotted line; p110δ inhibitor treatment.


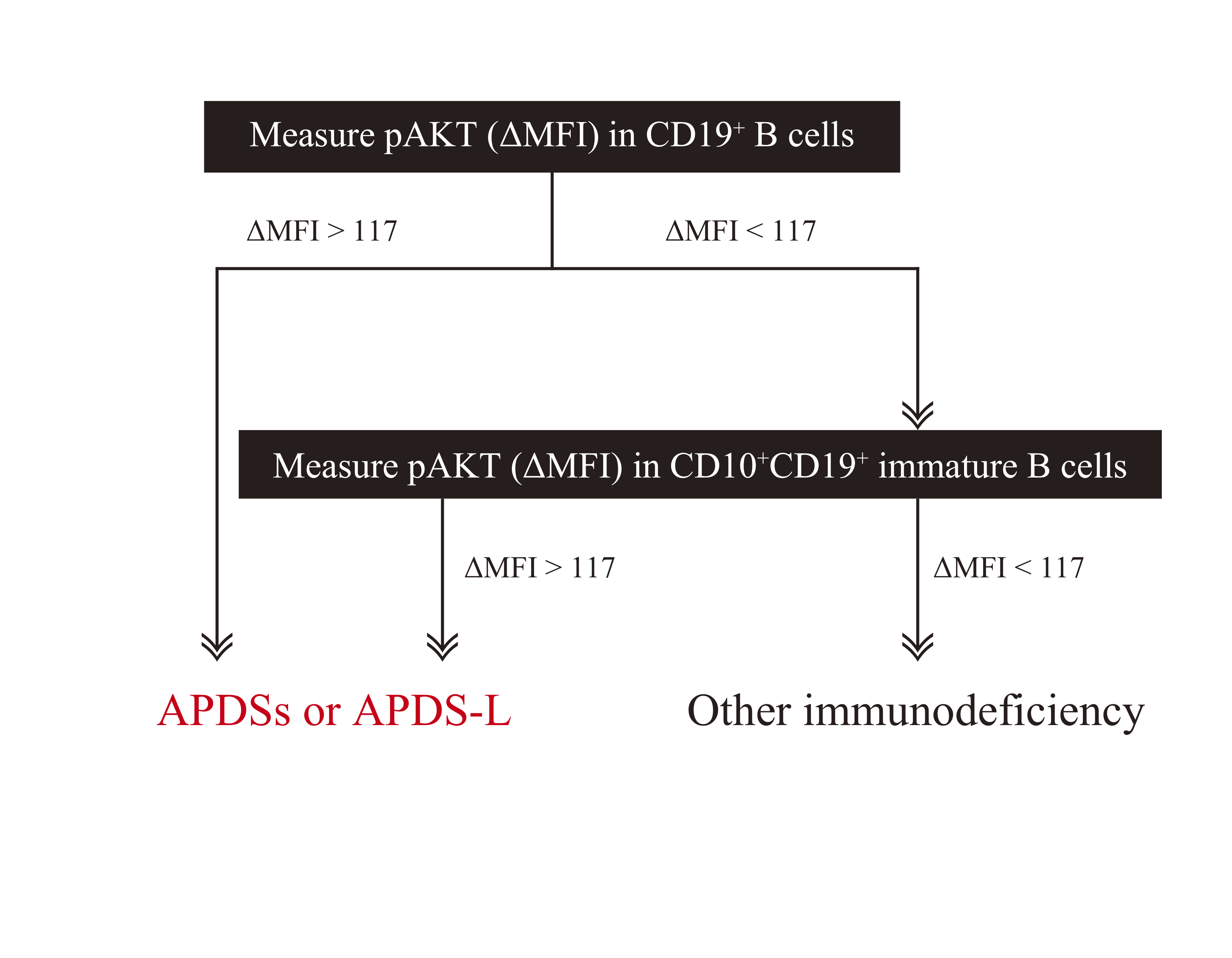


**SUPPLEMENTAL FIGURE 7**

**Flowchart for the rapid diagnosis of APDSs and APDS-L**

A flowchart of the flow cytometry-based rapid diagnostic test for APDSs and APDS-L used in the current study.

**
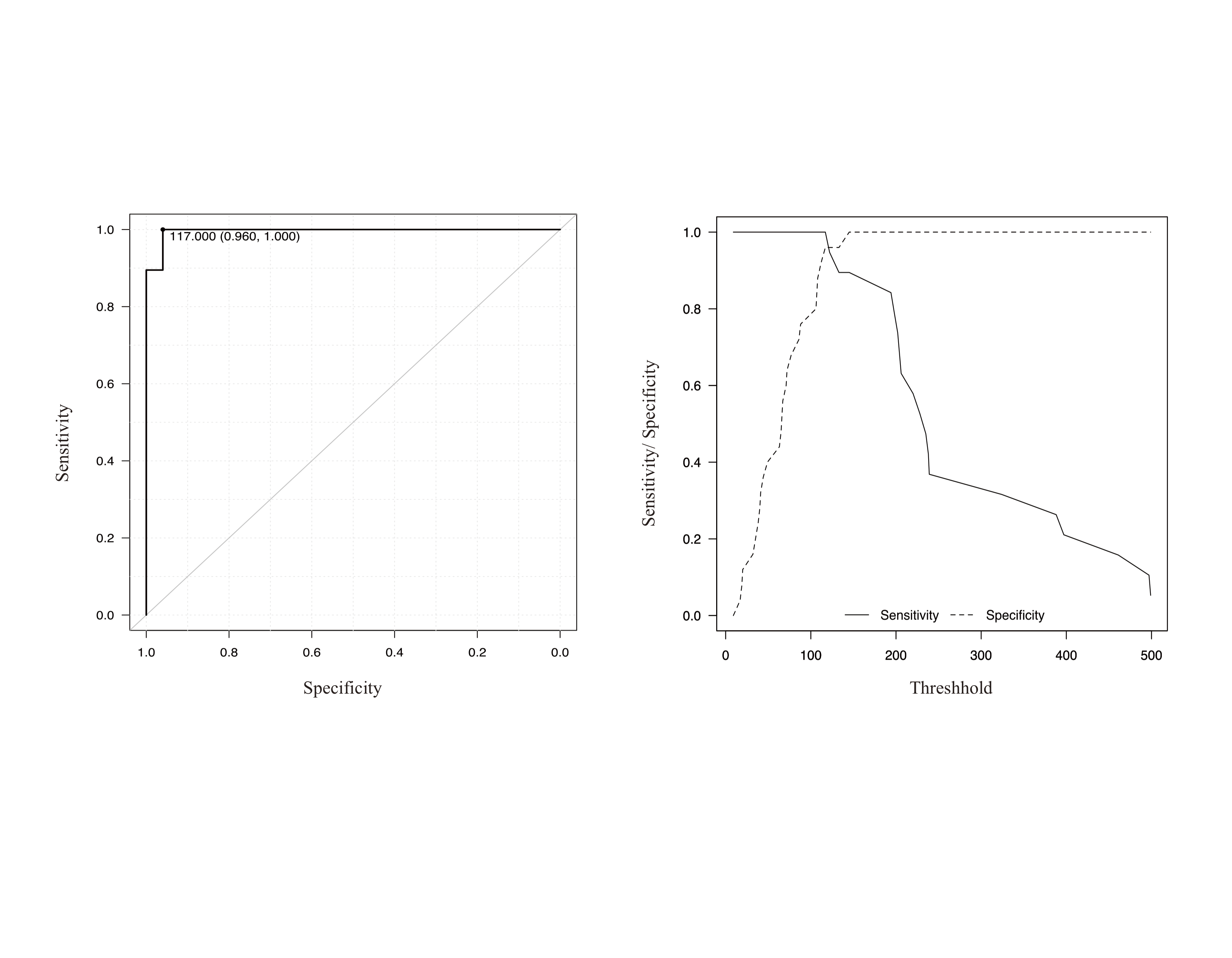
**

**SUPPLEMENTAL FIGURE 8**

**ROC curve**

Analysis was performed with EZR to create an ROC curve. The rate of specificity was 96.0%, and the sensitivity was 100%. The AUC was 0.996 (95% CI 0.986–1.000).

**SUPPLEMENTAL MATERIALS AND METHODS**

**Patients (clinical and immunological features)**

P1 (Family A)

P1 was an 18-year-old man of Japanese origin (Supplemental figure 1). He presented with recurrent episodes of pneumonia, otitis media, and lymphadenitis from the age of 2 years. At the age of 5 years, he developed antibiotic refractory pneumonia. Serum CMV specific IgG and IgM were positive, suggesting that he was in the acute phase of CMV infection. Simultaneously, he had hypogammaglobulinemia. He was thus diagnosed with CVID and started intravenous immunoglobulin (IVIG) treatment and prophylaxis with oral penicillin G and clarithromycin. At the age of 6 years, he developed mild lymph node swelling at the cervical and inguinal region. The histopathological examination of inguinal lymph nodes showed reactive follicular hyperplasia without the findings of lymphoma. We monitored the lymph node swelling without treatment, but no obvious progression or regression was observed. Thereafter, the patient experienced recurrent bacterial infections, regardless of treatment with IVIG or prophylaxis. A genetic study was performed at 16 years of age. A heterozygous mutation, c.1425+2 T>A, in *PIK3R1* was identified by whole exome sequencing (WES) and was confirmed by Sanger sequencing (Figure 1A, C). He was thus diagnosed with APDS2. The same mutation was not identified in his mother. No biological material was available from the other family members. Currently, he has short stature (−3.2 SD), mental retardation, enuresis, and hearing loss. He does not suffer from any symptoms that would suggest an autoimmune disorder or malignancy.

P2 (Family B)

The patient was a 19-year-old Japanese boy (Supplemental figure 1). At the age of 7 years, he presented with tonsillar enlargement and underwent tonsillectomy. The pathological diagnosis of the extracted tissue was diffuse large B cell lymphoma. He was started with chemotherapy. At the same time, he was given a diagnosis of HIGM (serum IgG = 281 mg/dl, IgA = 3.2 mg/dl, IgM = 294 mg/dl), and was started on periodic IVIG. At the age of 14 years, he developed mucosa-associated lymphoid tissue lymphoma in the right parotid gland, and was treated with chemotherapy. A genetic test was performed at 17 years of age. A heterozygous splice site mutation, c.1300-1 G>C, in *PIK3R1* was identified by WES and confirmed by Sanger sequencing. This mutation was not identified in his parents, suggesting that it was a *de novo* mutation.

P3 (Family C)

The patient was a 21-year-old Japanese man (Supplemental figure 1). He presented with recurrent bacterial infections, including otitis media, tonsillitis, phlegmon, and pneumonia from the age of 1 year. He then started to develop recurrent Quincke’s edema and warts with phlegmon from 11 years of age. He was diagnosed with IgG2 and G4 subclass deficiency with low IgA level at 12 years of age. He started IVIG treatment. At the age of 13 years old, he presented with cervical lymph node adenopathy and massive enlargement of the tonsils leading to laryngeal obstruction, and diagnosed as CMV lymphadenitis. He developed a laryngeal papilloma at 15 years of age. WES was performed at the age of 19 years. A heterozygous mutation, c.1425+1 G>C, in *PIK3R1* was found and confirmed by Sanger sequencing. This mutation was not identified in his parents, suggesting that it was a *de novo* mutation.

P4 (Family D)

The patient was a 4-year-old Japanese girl (Supplemental figure 1). She started to suffer from recurrent otitis media and bronchitis from the age of 2 months. At the age of 3 years, she was diagnosed with submandibular and inguinal lymphadenopathy. Histopathological examination of her axial lymph node showed reactive follicular hyperplasia without the findings of lymphoma. She had elevated IgM (542 mg/dL), low IgG2 (28 mg/dL), and positive results of QFT-3G. She suffered from progressive systemic lymphadenopathy, short stature, and delayed speech development. Although pathogenic mycobacterium was not isolated from clinical samples, she started treatment with streptomycin, isoniazid, rifampicin, and pyrazinamide under the suspicion of tuberculosis. Simultaneously, she was diagnosed as primary immunodeficiency and started IVIG treatment. An immunological examination before IVIG therapy showed a lack of isohemagglutination reaction and the poor production of measles-specific IgG antibody despite having a history of measles vaccination. She also had decreased numbers of CD19^+^ B cells and increased transitional B and follicular T cells in the peripheral blood. She was thus suspected to have APDS1, and a heterozygous mutation, c.1425+1 G>T, in *PIK3R1* was identified by a candidate gene approach. This mutation was not identified in her parents, suggesting that it was a *de novo* mutation.

**CVID and HIGM Patients summary**

**P5 (CVID)**

The patient was a 33-year-old Japanese male. From the age of 10, he has presented with recurrent otitis media 3 or 4 times per year. At the age of 19, he developed severe pneumonia. At the age of 25, he was diagnosed with severe hypogammaglobulinemia without the absence of B cells, requiring IVIG and leading to the diagnosis of CVID. Although WES was performed, the known PID gene was not detected.

**P6 (CVID)**

The patient was a 36-year-old Japanese man. From the age of 3, he has presented with recurrent otitis media. By the age of 12, he contracted pneumonia 5 times. At the age of 12, he was diagnosed with severe hypogammagloblinemia without the absence of B cells, requiring IVIG and leading to diagnosis of CVID. The absence of pathogenic mutations in *PIK3CD*, *PIK3R1* and *PTEN* has been confirmed by genetic sequencing.

**P7 (CVID)**

The patient was a 17-year-old Japanese girl. From the age of 13, she has presented with recurrent pneumonia and otitis media. At the age of 14, she was diagnosed with severe hypogammagloblinemia without the absence of B cells, requiring IVIG and leading to diagnosis of CVID. She suffers from bronchiectasis. Although WES was performed, the known PID gene was not detected.

**P8 (CVID)**

The patient was a 30 year-old Japanese woman. From the age of about 20, she has presented with several episodes with bacterial infections. At the age of 27, she developed severe pneumonia. Simultaneously, she was diagnosed with severe hypogammagloblinemia without the absence of B cells requiring IVIG. Thus, she was diagnosed with CVID. Although WES was performed, the known PID gene was not detected.

**P9 (HIGM)**

The patient was a 41-year-old Japanese man. From childhood, he has presented with recurrent otitis media and developed pneumonia generally twice per year. At the age of 39, he developed thoracic empyema. The elevated level of serum IgM and low level of serum IgA and IgM were detected. A genetic test identified a known hemizygous mutation, c. 761 C>T, in *CD40LG*, leading to a diagnosis of HIGM.
